# Brain metastasis effectively treated with erlotinib following the acquisition of resistance to gefitinib: a case report

**DOI:** 10.1186/1752-1947-8-64

**Published:** 2014-02-20

**Authors:** Sayaka Ohara, Tomonori Ushijima, Mariko Gunji, Chiharu Tanai, Yoshiaki Tanaka, Hiromichi Noda, Hajime Horiuchi, Kazuhiro Usui

**Affiliations:** 1 Division of Respirology NTT Medical Center TOKYO, 5-9-22 Higashigotanda, Shinagawa, Tokyo 141-8625, Japan; 2 Department of Diagnostic Pathology, NTT Medical Center Tokyo, 5-9-22 Higashigotanda, Shinagawa, Tokyo 141-8625, Japan

## Abstract

**Introduction:**

Non-small-cell lung cancer harboring an activated epidermal growth factor receptor mutation exhibits a good response to epidermal growth factor receptor-tyrosine kinase inhibitors; however, clinicians often experience treatment failure following the development of resistance to epidermal growth factor receptor-tyrosine kinase inhibitor.

**Case presentation:**

We here report a case of a 56-year-old Japanese woman with non-small-cell lung carcinoma with a secondary T790M mutation associated with resistance to epidermal growth factor receptor-tyrosine kinase inhibitor that maintained sensitivity of brain metastases to epidermal growth factor receptor-tyrosine kinase inhibitor. An autopsy showed that the primary focus had a T790M mutation; however, no mutations of T790M were found in the brain metastases.

**Conclusion:**

This case demonstrates the detection of T790M was associated with the clinical responsiveness to epidermal growth factor receptor-tyrosine kinase inhibitor.

## Introduction

Gefitinib and erlotinib are epidermal growth factor receptor-tyrosine kinase inhibitors (EGFR-TKIs). A non-small-cell lung cancer (NSCLC) with an EGFR mutation exhibits a good response to EGFR-TKI [1,2].

Despite initially showing a good response, most patients present with recurrence of the disease; this is due to the development of resistance to EGFR-TKI. Several mechanisms of acquiring resistance to EGFR-TKI have been reported. The existence of a second mutation of T790M [3,4], amplification of c-met [5,6] and overexpression of hepatocyte growth factor (HGF) [7] are known mechanisms of developing resistance to EGFR-TKI.

No standard treatment for patients who exhibit disease progression during EGFR-TKI therapy has been established. Clinicians sometimes experience cases in which, although EGFR-TKI is unable to maintain disease control of the primary focus, the drug is effective against brain metastases.

We here report an autopsied case of NSCLC with an EGFR mutation treated with gefitinib that resulted in the progression of the disease while maintaining EGFR-TKI’s effectiveness against brain metastases. We analyzed the status of the EGFR mutation in the primary site and brain metastases using a high-sensitivity method, the peptide nucleic acid-locked nucleic acid polymerase chain reaction (PNA-LNA PCR) clamp method [8].

## Case presentation

**Figure 1:**
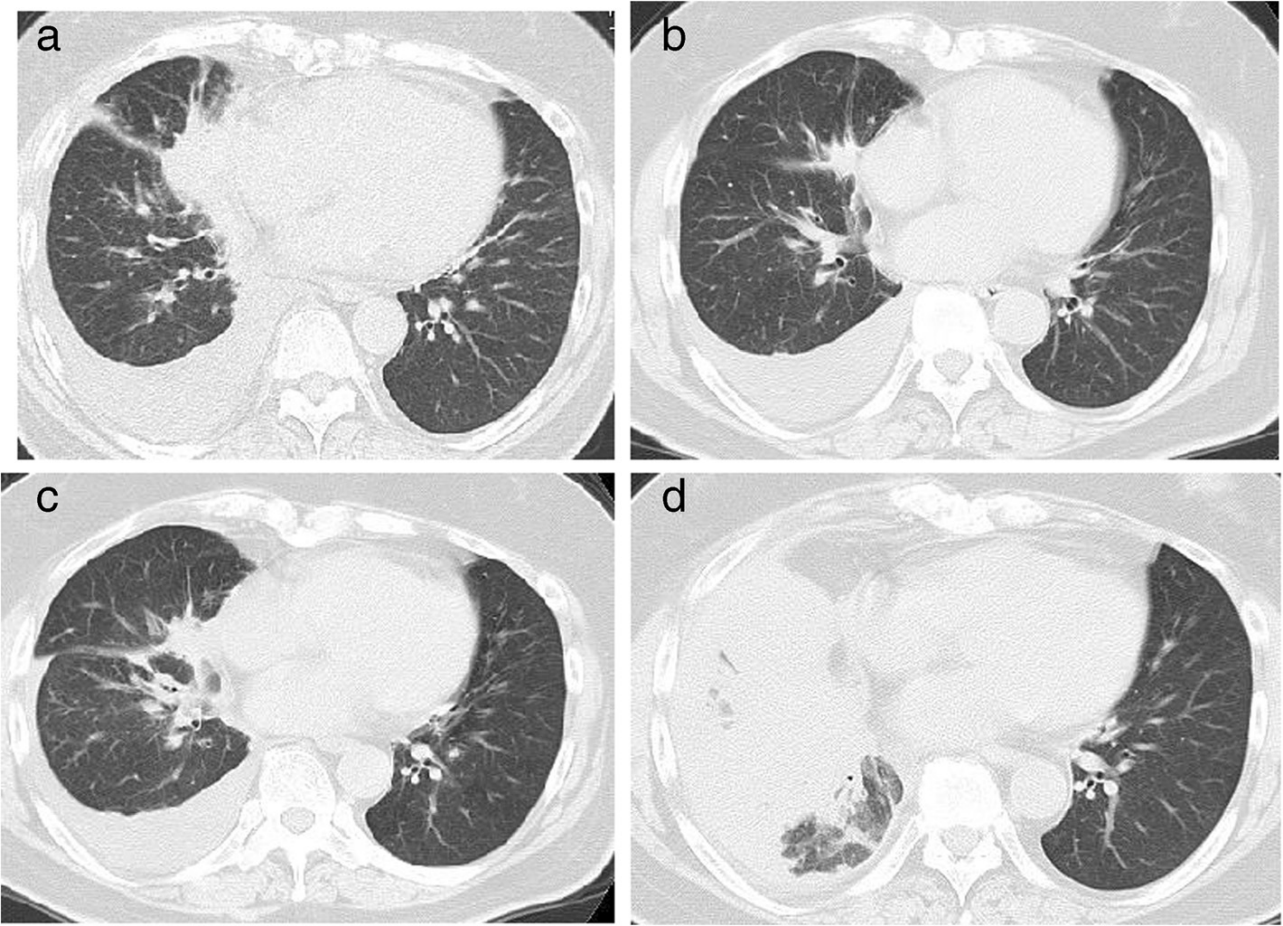
**Computed tomography scans. a)** On the initial medical examination, right pleural effusion and a mass in the middle lung field were observed. **b)** After 3 months of gefitinib treatment, an improvement in the right pleural effusion and a decrease in the size of the primary lesion were observed. **c)** After 5 months of gefitinib treatment, progression of the size of the mass was observed. **d)** Infiltration and atelectasis of the right lung were observed.

A 56-year-old Japanese woman with a previous history of cigarette smoking was referred to our hospital in September 2010 for a cough. Computed tomography (CT) showed pleural effusion in her right thoracic cavity with a tumor in the right middle lobe (Figure 1a). Cytology of the pleural fluid revealed adenocarcinoma with an EGFR mutation of an exon 19 deletion without a T790M mutation. Clinical stage IV (cT4N3M1a) adenocarcinoma was diagnosed.

The first-line treatment was gefitinib, an EGFR-TKI, which resulted in a partial response for 3 months (Figure 1b). However, after 5 months of gefitinib treatment, we observed an increase in her right pleural effusion in March 2011. Repeat cytology of her right pleural fluid showed adenocarcinoma with an EGFR exon 19 deletion and a second mutation of T790M associated with EGFR-TKI resistance.

**Figure 2:**
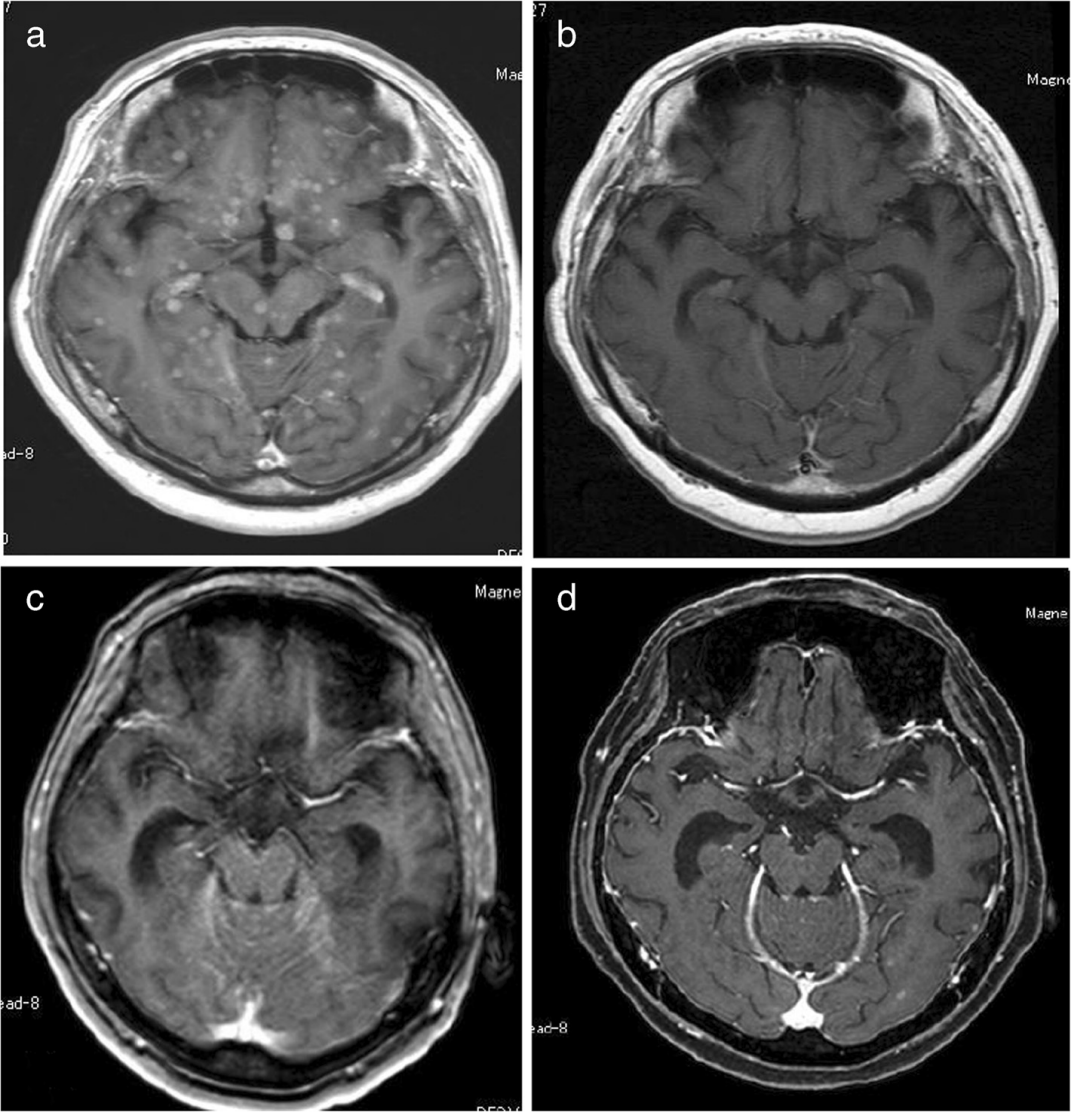
**Brain magnetic resonance imaging (T1 gadolinium). a)** Multiple, enhanced miliary nodules were observed. **b)** Following whole-brain radiotherapy and erlotinib treatment, the complete disappearance of the metastatic lesions was observed. **c)** Following docetaxel, an intensive signal in the cisterna of the posterior cranial fossa indicating carcinomatous meningitis was observed. **d)** After 1 month of the rechallenge of erlotinib treatment.

We introduced cytotoxic chemotherapy. After five cycles of carboplatin-pemetrexed, a relapse in her right pleural effusion was noted (Figure 1c). After pleurodesis, followed by four cycles of gemcitabine, the patient complained of disorientation in October 2011. Despite the primary lesion being well controlled, brain magnetic resonance imaging (MRI) with gadolinium-diethylene-triamine pentaacetic acid showed miliary brain metastases (Figure 2a).

The patient received whole-brain radiotherapy (30Gy/10 fractions) until the end of October 2011. The fourth-line treatment was erlotinib, which was started at the end of October 2011.

The erlotinib treatment was continued until February 2012. By that time, her complaints of disorientation had resolved, and brain MRI showed disappearance of her brain metastases (Figure 2b). However, a chest CT showed disease progression of the primary lesion, and her right lung was almost in a state of atelectasis (Figure 1d).

We terminated the erlotinib treatment and initiated therapy with docetaxel.

In April 2012, she was hospitalized for inability to walk and depressed level of consciousness, brain MRI showed carcinomatous meningitis (Figure 2c). Although she could hardly eat, a rechallenge of erlotinib treatment was undertaken.

After rechallenge of erlotinib, although there was no improvement in her ability to walk, her level of consciousness improved a little, and she could eat more. Brain MRI demonstrated an improvement in the carcinomatous meningitis in May 2012, 1 month after the rechallenge of erlotinib treatment (Figure 2d). An improvement in clinical symptoms and radiological findings show that erlotinib treatment was effective.

However, her general status worsened, and she died of dyspnea in July 2012.

An autopsy was performed after the patient’s death. The autopsy showed that her right lung was almost entirely infiltrated by the tumor. Direct invasion of the tumor into her thoracic wall, pericardium and diaphragm was seen. There were multiple metastases in both lungs and her liver, right adrenal gland, peritoneum, brain, spinal cord, meningeal and lymph nodes.

Although brain metastasis was found at autopsy, the gross pathology of her brain showed few metastases. Many small, yellow nodules were found on her brain surface; however, they resulted from invasion of foam cells, indicating traces of metastases treated by previous chemotherapy.

**Figure 3:**
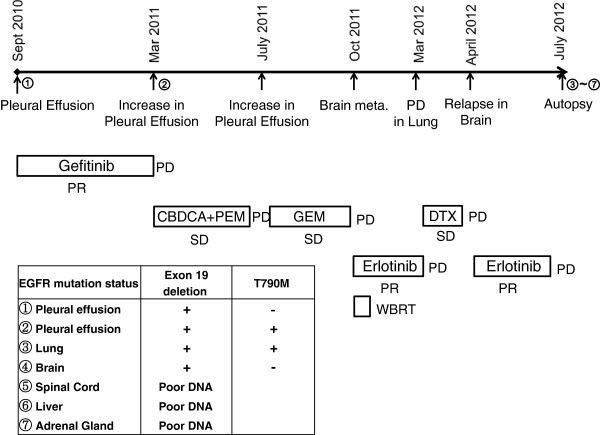
Chronology of the disease progression, treatment and epidermal growth factor receptor mutation status.

The EGFR mutation in the primary lesion and brain metastases was reassessed using the PNA-LNA PCR clamp method. An EGFR mutation of an exon 19 deletion was found in both the primary lesion and brain metastases. Cytology showed the development of right pleural effusion after gefitinib treatment, and a second mutation of T790M was found in the primary lesion. However, the brain metastases exhibited no mutations of T790M (Figure 3).

## Discussion

Here we reported the autopsy case of a patient whose brain metastases responded well to erlotinib, even after acquiring resistance to EGFR-TKI at extracranial sites. An analysis of the EGFR mutation using a PNA-LNA PCR clamp method showed that no resistant mutations (T790M) were detected in the sites of brain metastasis, although this mutation was detected in the extracranial sites. The detection of T790M was associated with the clinical responsiveness to erlotinib in this case.

Among the reported patients with NSCLC, approximately 20% to 40% develop brain metastases at some point [9,10]. Several reports have suggested the possibility that EGFR-mutated tumors are prone to brain dissemination [11,12]. Omuro *et al.* reported that the central nervous system (CNS) is a frequent site of disease recurrence in patients with NSCLC following an initial response to gefitinib, regardless of disease control in the lungs [11]. Park *et al.* reported that metastatic brain tumors in patients with NSCLC harboring EGFR mutations respond well to EGFR-TKIs as a first-line treatment and that the type of the EGFR-TKI used has no effect on progression-free survival or overall survival [13]. Controlling brain metastasis is important in patients with NSCLC with EGFR mutations.

Jackman *et al.* reported a case of a patient with NSCLC harboring EGFR mutation in which high-dose gefitinib was needed to achieve cerebrospinal fluid (CSF) drug levels that were adequate enough to cause tumor growth inhibition [14]. In addition, the case indicates that extended gefitinib exposure in the systemic lung cancer sites was associated with the development of a systemic T790M resistance mutation, whereas CNS metastases did not undergo this secondary change.

Ruppert *et al.* reported a case of a lung adenocarcinoma with a sensitive EGFR mutation in which the patient developed brain metastases with carcinomatous meningitis and liver metastasis after he stopped taking EGFR-TKI [15]. Since a secondary T790M mutation was found on a liver biopsy, but not in the CSF, the patient received erlotinib, and the brain metastasis responded well to treatment. As in that case report, our patient exhibited sensitivity to erlotinib in brain metastases, even though the cancer cells in the pleural effusion had acquired a resistant mutation to EGFR-TKI, T790M. An autopsy analysis using the PNA-LNA PCR clamp method showed that, although a sensitive mutation to EGFR-TKI (exon 19 deletion) was found in both the primary lesion and brain metastases, a resistant EGFR mutation (T790M) was found only in the primary site. In our case, as the disease progressed to the patient’s CNS, applying cytotoxic chemotherapy became difficult because of her worsened general status. In such a case, although she died of progressive disease of T790M mutated tumor cells in her thorax, EGFR-TKI, which was effective in the brain metastases, was beneficial for her to improve her quality of life.

These cases show that the possibility of persistent cerebral TKI sensitivity should be considered in patients presenting with CNS relapse after stopping an EGFR-TKI, even those with a T790M-resistant mutation in non-cerebral metastases. We examined the EGFR mutations in the tumors in the lungs and brain only. The status of the EGFR mutation was examined using the PNA-LNA PCR clamp method; however, there are technical limitations in the ability of this test to detect mutations. To help us understand why T790M was not found in the brain metastases, we tried to investigate the EGFR mutation status in other metastatic sites such as the liver, adrenal gland and spinal cord. However, because of the technical limitation of fixation of formalin of an autopsy case, we could not extract adequate deoxyribonucleic acid (DNA) to investigate their EGFR mutation status.

Other types of resistance, such as met amplification and/or HGF overexpression, have different effects on brain metastases. Although these limitations exist, the different distribution of the cancer cells that had acquired the T790M mutation primarily explains the different response to erlotinib observed between the intracranial and extracranial sites in this case.

## Conclusions

It is worth administering EGFR-TKI in patients with brain metastases, even when the patient acquires EGFR-TKI resistance. Based on the status of the EGFR mutation in critical sites (particularly the CNS), clinicians should decide whether to continue, stop or conduct a rechallenge of EGFR-TKI when the disease progresses. Further investigations as to why resistance to EGFR-TKI appears and exhibits a different distribution are necessary in order to provide better treatment to patients with NSCLC with EGFR mutations.

## Consent

Written informed consent was obtained from the patient for publication of this case report and accompanying images. A copy of the written consent is available for review by the Editor-in-Chief of this journal.

## Abbreviations

CBDCA: carboplatin; DTX: docetaxel; EGFR: Epidermal Growth Factor Receptor; GEM: gemcitabine; PEM: pemetrexed; WBRT: whole-brain radiotherapy; meta: metastasis; PD: progressive disesase; PR: partial response; SD: stable disease; DNA: deoxyribonucleic acid.

## Competing interests

The authors declare that they have no competing interests.

## Authors’ contributions

CT, YT and HN were members of the Division of Respirology and contributed not only in the treatment of the patient, but also in analyzing and interpreting the case. TU, MG and HH were members of the Division of Diagnostic Pathology and performed the autopsy of the case and analyzed EGFR mutation status. KU was the physician in charge of the patient and interpreted the clinical data and autopsy report. SO was an attending physician of the patient, and analyzed and interpreted the clinical data and autopsy report, and was a major contributor in writing the manuscript. All authors read and approved the final manuscript.
